# Betrayed, Beaten, Banished: The Stigma of Being a Rural *Tongqi* in China

**DOI:** 10.3390/ijerph21091125

**Published:** 2024-08-26

**Authors:** Eileen Y. H. Tsang, Fang Yueyao

**Affiliations:** Department of Social and Behavioral Sciences, City University of Hong Kong, Hong Kong 852, China; yueyafang2-c@my.cityu.edu.hk

**Keywords:** enacted stigma, extended family, patrilineal marriage, *Tongqi*

## Abstract

In China, an emerging social issue involves a subset of rural women who, because of family and culture, become inadvertently matched up with and married to closeted men who have sex with men (MSM). These women—referred to as *Tongqi*—often discover they are in a loveless marriage, but any effort to change their situation results in intense backlash, discrimination, and stigma from families, village communities, and even government and healthcare institutions. This study explores the experiences of *Tongqi*, examining the influence of social interaction, community relationships, and macrostructural factors that coalesce to create an environment of chronic enacted stigma. In-depth interviews were conducted with 59 rural *Tongqi*, 11 of whom contracted human immunodeficiency virus (HIV) or acquired immunodeficiency syndrome (AIDS) from their spouses. The findings reveal the significant role of extended kinship networks and macrostructural elements, such as hukou (household registration) and government officers, as well as village-level lineage structures. Informant data highlights how lineage relationships, interwoven with gender practices, contribute to the enacted stigma impacting the physical and psychological health of *Tongqi. Tongqi* report psychological effects such as an array of symptoms reflecting post-traumatic stress, chronic depression, and attempted suicide. *Tongqi* also report adverse physical health concerns involving reproductive health, unwanted pregnancies, sexually transmitted infections (STIs), and pregnancy complications. These findings helped produce possible policy recommendations to address the most pressing issues faced by *Tongqi*.

## 1. Introduction

The experiences of *Tongqi* (同妻) in rural China are encapsulated, highlighting their struggles with their MSM husbands’ families, fellow villagers, and some macrostructural factors. *Tongqi* (literally translated as “homowives” 同妻) is the term used to describe heterosexual women who find themselves paired or matched in marriage to MSM. These women often unwittingly enter into fake marriages and do not learn about their situation until they contract STIs.

Such a case occurred on the morning of 15 July 2019, in the small village of Jilin in Heilongjiang Province. Shanni, a 29-year-old *Tongqi*, recounted her ordeal at the hands of her in-laws, siblings of the husband’s family, and, of course, some local villagers.

Shanni (29) captured the altercation on her smartphone and showed it to me. The video revealed a heated argument between herself, her mother-in-law, and her husband, who was outed several weeks earlier. The fight began when the mother-in-law refused to stop criticizing her about household chores, calling her lazy and a lousy wife. At that moment, her husband came home and joined his mother, calling her lazy, stupid, and selfish. As Shanni began to argue back, the husband and his mother physically attacked Shanni in the living room, grabbing her and slapping her face. Although she fought back, she was overpowered and subdued.

The commotion was so loud that two male villagers and three women approached the door. When they saw what was happening, they joined in to restrain Shanni alongside her husband and mother-in-law. In the video, Shanni’s mother-in-law is visibly agitated, repeatedly pushing her, calling her names, and cursing. Yet another male villager reached in and grabbed Shanni’s hair once more. Overwhelmed by the group of five, Shanni appeared defeated and helpless. Desperate, she raised her voice, crying out for assistance. Her pleas attracted more villagers, both men and women, exacerbating the chaotic situation. But rather than intervening to help, they watched and recorded video of the scene on their smartphones.

This article investigates the marriages of 59 heterosexual women to MSM in rural China. The concept of enacted stigma, or unfair treatment by others, anchors the theoretical framework. Enacted stigma is commonly used to describe discrimination in all its forms. This study examines how these women cope with multiple layers of hostility and ill-treatment. *Tongqi* face harassment and bullying from their husbands’ families as well as neighbors and villagers where they live [1]. The interactions between *Tongqi* and their male partners, as well as their engagement with family, community, local government bureaucracies, and broader national rules and laws, such as those related to the hukou system, are intertwined and complementary to create an environment of chronic punishment. The ensuing analysis navigates through various layers or dimensions of stigma, reflecting systematically on the mutual implications and interactions between these dimensions. Social structures, including class and gender, shape societal norms and behaviors [2]. Enacted stigma, referring to tangible discrimination due to non-conformance, is typically theorized outside the symbolic interactionist framework [3].

Therefore, the research inquiries presented here are: (1) In what ways do in-laws, MSM’s siblings, and villagers within a patrilineal marriage enact stigma against *Tongqi*? (2) How do institutional factors such as hukou and government employment exacerbate enacted stigma experienced by *Tongqi*? These research inquiries are addressed in the following four sections. Section 1 presents the conceptual framework detailing how day-to-day family interactions coalesce with macrostructural factors to create an environment of chronic enacted stigma against *Tongqi*. Section 2 outlines the research methodology and the collection of ethnographic data from a specific sample of *Tongqi*, recruited with consent from their husbands and referrals from other *Tongqi* and non-governmental organizations (NGOs). Section 3 presents the data and subsequent analysis. The Conclusion recommends evidence-based policies such as counseling services, enhanced medical treatment for rural *Tongqi* who contract HIV, and ways to establish a supportive sisterhood using social media platforms.

## 2. Methods and Data

Data were collected over 36 months, from January 2019 to June 2023, in Shandong (Qingdao), Liaoning (Shenyang), Heilongjiang (Harbin), and Jilan (Changchun) provinces. Before the COVID-19 pandemic (January 2019–January 2020) and following the easing of COVID-19 restrictions in China from January 2023, all data were gathered through in-person interactions. However, during the pandemic (February 2020–December 2022), data were collected virtually, relying on referrals from *Tongqi* or MSM. Ethnographic interviews were conducted with *Tongqi*. Over the three years of data collection, 120 *Tongqi* were contacted using snowball sampling, where interviewees referred me to other *Tongqi* throughout northeastern China. Of the 120 *Tongqi* initially contacted, 59 agreed to be interviewed. All of the participants came from rural China and were between the ages of 29–40. Only four did not have a child; the rest had at least one child. Five reported having no formal education, while the other 54 only attended primary school. Although none of the *Tongqi* or MSM involved in the study reported they were part of an arranged marriage, this distinction has to do with that definition. To these participants, an arranged marriage refers to a union where a couple first meets at the wedding itself. The parents made all the decisions in advance with the consent of either the son/husband or daughter/wife. All the participants in this study had met at least once before getting married, either online via WeChat or through family, friends, and acquaintances. Notably, after marriage, 11 *Tongqi* admitted they had contracted HIV from their husbands. They were taking medications, and their health situation was satisfactory.

This research went through three iterations in arranging interviews. An initial set of interviews with the 101 MSM from a previous project resulted in the request for follow-up with their wives. MSM were former male sex workers, village cadres, military service, taxi drivers, factory workers, and other blue-collar workers. The 101 men had been outed to their wives and gave the investigator permission to contact them, resulting in 32 interviews; another 13 *Tongqi* were interviewed through the 12 LGBT non-governmental organizations in Shandong (Qingdao), Liaoning (Shenyang), Jilin (Changchun), and Heilongjiang (Harbin). An additional 14 *Tongqi* were recruited and interviewed through personal and professional networks. All 59 interviews were conducted before the global pandemic outbreak and followed up afterward with WeChat to communicate with them during the pandemic. All participating *Tongqi* remained in their marriage relationships, and the average duration of their marriages was around six years.

Travel to participants’ villages enabled first-hand observation of *Tongqi* in their homes, communities, and the public spaces discussed in their narratives. Exploring and sharing sensitive memories of highly stigmatized bodily commodification was challenging. The study’s purpose was explained to ensure the participants’ privacy and the findings’ accuracy. Participants signed informed consent forms and were notified of their right to withdraw from the study voluntarily at any time. Upon completion of the interview, each participant received CNY 180 (around USD 30) for their participation.

Qualitative, flexible, interactive, and semi-structured interviews elicited in-depth information from participants that described intimate aspects of the informants’ lives. Therein, the informants provided detailed descriptions that help the reader understand and identify broader concepts and perspectives. In the present study, the assembly and organization of the research questions guided the interview and covered the study’s core themes.

The interviews included questions such as “How and when did you discover your husband is MSM? How did that discovery impact your interactions with your husband’s family, his work colleagues, and friends in the village? What types of discrimination or types of conflicts have you encountered since discovering his sexual orientation? How did you cope mentally and emotionally with the discrimination and conflict you began to experience? In your view, how does the extended family structure and rural hukou affect you as a *Tongqi*? How did contracting HIV affect your mental health? For all the conflict and discrimination you experience, what keeps you in this marriage relationship?”

Informal follow-up data collection methods like QQ or WeChat were helpful because the global pandemic restricted travel amid health concerns. Therefore, face-to-face interviews, participant observation, and online engagement through social media enhanced the data’s depth through direct knowledge of the informants’ daily activities and life histories [4].

The accumulated data comprised recorded interviews, in situ note-taking, and post-event field notes. Each interview was transcribed, translated into English from Putonghua, the informant and researcher’s native language, and uploaded into NVivo 11.0. The main conceptual framework themes guided the initial analysis, which generated additional themes that were categorized and re-coded. The coding was inspired by grounded theory [5] since little has been written about the stigma of *Tongqi* in China. The data from earlier interviews were coded, and follow-up questions were conducted through WeChat. In this analysis, there was a priori consideration of the arena in which stigma would occur involving the partner, family, and villagers. Stigma was explored in follow-up interviews through questions about structural factors such as the villagers, society, and their relationships with the MSM and their families. The process enabled additional significant themes to emerge.

An open coding approach to thematic analysis allows smaller categories to be aggregated, producing broad themes regarding mental health and sociocultural factors contributing to stigma [6]. These themes were aggregated and used to theorize and interpret the data. Results were then analyzed again for sub-themes such as domestic violence, stigma, and patrilineal families. Stigma and health emerged as the central theoretical theme. In the current study, axial coding compared and categorized codes and identified subordinate themes. Selective coding comparisons generated superordinate themes to develop the finding’s theoretical implications.

This article used pseudonyms and slightly modified biographies to mask the participants’ identities. The translation from Chinese to English sought to maintain the spirit and intention of the informants’ viewpoints. The Institutional Review Board approved the study at the researcher’s host university.

### Background of Tongqi in China

*Tongqi* are rarely equipped to defend themselves from various attacks led by their MSM. Although there is no official data, it is estimated that in 2022, there were approximately 25 million women in rural areas of China who unknowingly married closeted MSM [7]. Often, these women discover their husbands’ secret shortly after giving birth. Once the couple has fulfilled their filial and marital obligations to produce a baby, sudden and drastic behavioral changes may occur as the MSM ceases to be physically intimate. Of the estimated 25 million *Tongqi*, approximately 14 million *Tongqi* find themselves facing discrimination [8] and being ostracized as a result of a conventional ancestral gendered family system that pressures MSM to enter a heterosexual union for ancestry inheritance. In China, *Tongqi* have been portrayed as victims of marriage fraud who are essentially ‘living widows’ trapped in a sexless and loveless marriage. They are also at risk of adverse physical, mental, and emotional consequences [9]. The study participants in northern China struggled economically, reporting their approximate annual income of USD 1900 (CNY 12,000).

Unable to exercise agency “in the interstices of power” [10], an MSM will marry a *Tongqi* to have a child and fulfill the filial son obligations to his family to meet the expectations of his relatives and even the village. For young women, there is enormous pressure to marry, give birth, and avoid becoming a “leftover lady” (剩女). The derogatory term refers to single women older than 27 years old [7]. Being a leftover lady is a derogatory term that is still considered a form of humiliation in rural China. Parents—particularly mothers—go to public parks to negotiate arrangements to bring the unmarried sons and daughters together. In rural China, leftover ladies often face negative stereotypes, being perceived as overly selective, socially undesirable, and subjected to stigmatization. They are still viewed as incomplete or unsuccessful if they are not married. This leads some to feel pressured to “sell themselves” in the marriage market, presenting themselves as potential wives to potential suitors.

*Tongqi*, by contrast, are often bereft of financial independence. For the few who may have financial resources, the legal system itself carries a clear gender bias favoring men in marital disputes. The adjudication of women’s divorce claims presents formidable challenges, and even those women who manage to secure a divorce often encounter obstacles in obtaining the compensation to which they are entitled.

## 3. Findings

### 3.1. Enacted Stigma from In-Laws or Husbands’ Siblings

All 59 interviewees were married to MSM and encountered enacted stigma from their in-laws and husbands’ siblings. This stigma is linked to traditional expectations for women to bear sons [11]. For example, Ziwei, aged 31, contracted HIV from her husband. She married in 2011 and gave birth to a daughter the following year. However, her mother-in-law wanted them to keep trying until they produced a son. However, Ziwei noticed that her husband kept several forms of lubricating oil and antiretroviral medication in their washroom. When she questioned him about it, he admitted he was MSM. The revelation stunned Ziwei. Shortly after that, she found she had contracted AIDS from her husband. In her despair, she said the only thing that kept her going was her love for her daughter, who had autism. Ziwei reported that her mother-in-law regularly pushed, slapped, and punched her. She recalled what her mother-in-law once said as she scolded her:

“You married my son because you want a man who can buy you things, feed you, and support your family. But what did you give him? A daughter? My son gave money to your poor family in Shenyang. Don’t ever think you are a queen or a princess. You are only a leftover lady. Who wants an old woman? My son was so generous to pick you up, like he saved the environment by removing trash from a landfill. If you are unhappy, leave this village and find your dream man”. Then, I was beaten with a metal broom. My spinal cord was injured, and I stayed in the hospital for 6 months…”

Qingwei (31) said that when she discovered her husband was MSM, they agreed to sleep in separate rooms. Their daughter had been diagnosed with Down’s syndrome, and it was left for Qingwei to raise their daughter and do all the household chores. Her father-in-law showed little sympathy and continually scolded her:

“With your inferior genes, of course, you can only produce a daughter and one who has Down’s syndrome. Our neighbors scoffed at me when I went to the paddy field. Do you know how I felt? AWFUL!” I know it is only an excuse to blame me, and they wanted me to leave the village. My in-laws did not want my daughter, but where could we go?”

All the *Tongqi* identified the mother-in-law as probably the most visible source of enacted stigma. Xiaotung (32) said her mother-in-law aggressively criticized her and blamed her for all their troubles more than any family member. She said:

“His whole family knew he was an MSM long before we were married. My mother-in-law criticized everything I did and did not like me at all. One day, she suddenly asked me why I was not seductive enough to change his sexual orientation. Later, he tested positive for HIV—and I found I had it too! She said: “You know, HIV will not make you die immediately, right? Probably, in your previous life, you did something wrong. Do not bring all these inauspicious things to our family and village. Take your things and get out!”

The rumor quickly spread across the village that Xiaotung (32) was not a good wife, mother, or daughter-in-law. In the village, she was not even attractive enough to change her husband’s sexual orientation. Tortured by her husband’s family as a salacious and irresponsible woman, Xiaotung suffered from chronic depression and thought of killing herself and her two young girls. She experienced hallucinations and heard voices in her head telling her to dump their children on a mountain, hide them in the forest, and bury them in the backyard. She saw a doctor and was diagnosed with schizophrenia. Her husband wanted to maintain his face. Her-in-laws took care of the children. She lived in the basement of the house, and her in-laws did not allow her to see her children.

Another *Tongqi*, Danyi (36), was married to her MSM for 5 years. She gave birth to a daughter during their first year together, but she could not conceive after that. Her husband worked at a factory in Guangdong, several hours and hundreds of kilometers away. Once a year, he would visit her, but only for a few days. She said:

“Every time my sister-in-law visited during holidays, she would not even say hi. She would bring fancy food and fruit but always found a way to insult me. “Your folks only bring us salty fish and potatoes. We give you everything, and you just have a daughter?” I knew they were even hiding food. Leftovers would disappear from the fridge, but I could still smell the strawberries. They treated me like an unwanted stepchild. I never felt welcome.”

Danyi (36) explicitly stated that patriarchal norms pervade her village. Historically, it has been customary for Chinese parents to prefer sons over daughters [12]. In the Chinese patrilineal family structure, sons are preferred because they can own property, perpetuate the family lineage, live with their parents and care for them in their twilight years, and perform ancestral rites.

Another *Tongqi*, Wangyue (31), did not produce a child after she married her MSM. Her husband did not want to touch her after they married. Her brother-in-law scolded her as a useless and stupid woman. She recalled what he said to her:

“You did not need to stay here and complain. You are only an egg machine to give birth to a child! However, you are a stupid woman without a womb to carry a baby. My brother needed to cut off his losses and dump you. However, he is still so kind and provides you food and shelter”. Then he would kick me, wrestle me on the floor, grab fistfuls of my hair, and shake me. I was too weak to fight back and almost knocked unconscious. When it was over and I resumed consciousness, I called 120 (emergency call) and spent one week in the hospital.”

Another *Tongqi*, Beibei (29), said her mother-in-law kept asking her to disappear and leave her family quietly. She was warned against filing for divorce, however. Her mother-in-law said,

“Your marriage is just a bad business deal. You know my son is MSM, so you can leave. Don’t stay, and don’t fight. This is an ultimatum: you will regret it if you try to divorce. You are a total failure. Don’t ever think you can compete with us; we will destroy you. Don’t ask our grandson to go to your poor village with you. You cannot even provide food and education for him. Shameful!”

The verbal attacks and threats continued, and Beibei finally went to the local police. But her complaint was dismissed immediately because there was no evidence. Among all *Tongqi* interviewed, the rural police were described as unhelpful, even negligent, toward their situations. Beibei (29) went home feeling hopeless and depressed. To the police, no physical evidence of injuries showed the incident was consensual. For example, the police asked Beibei (29) to show them pictures and videos of her in-laws beating her up. Police in China often consider domestic violence to be embodied by a woman’s willingness to sacrifice her life to protect her chastity and honor.

All the *Tongqi* in these rural settings consider themselves part of patrilineal marriages, adopting their husband’s surnames. Patrilineal marriage refers to a social system in which inheritance and family lineage are traced through the male line [12]. This system prioritizes male offspring to continue the family line and legacy of property. In this context, *Tongqi* must routinely endure traumatic experiences that are defined by exclusion and a lack of accountability for the perpetrators [13]. *Tongqi* have to live with their in-laws and MSM as second-class citizens, alone and under constant attacks.

### 3.2. Enacted Stigma Received from the Villagers

The *Tongqi* reported they often experienced verbal insults and sometimes physical assaults from villagers. They also faced disapproving silent stares from both male and female villagers. Patriarchal norms reinforce the roles of *Tongqi* as publicly submissive partners to their husbands, perpetuating a cycle of domination and subordination. In general, the villagers mostly ignored the distress of the *Tongqi*, only caring if the *Tongqi* used social media to identify themselves and their plight. They were primarily concerned about the image or reputation of the village if negative posts appeared on platforms like WeChat or shared with specific *Tongqi* non-governmental organizations (NGOs). Haoyi (39) experienced stigma from villagers. She said:

“The villagers always seemed eager to prepare snacks and drinks for the entertaining drama we regularly provided. Criticism became an argument that got louder and louder. Soon, someone is screaming that I am a poor idiot, a leftover lady who cannot afford a house or a divorce. They were even teasing me about my HIV infection from my husband. Those people never thought my husband was MSM! They believed I was a lonely loser who had an affair and a one-night stand, and that is how I contracted HIV! One neighbor always yelled: “We are ashamed of you and hope you pack and leave soon... you are deeply bound by the threads of fate (ming 命)”.”

In the context of rural village norms and patrilineal marriage, *Tongqi* are not permitted to disclose their problems online because that damages everyone’s reputation needlessly. A second reason is that *Tongqi* should remain silent because they already benefitted from the traditional practice of “bride price”. Traditionally, the groom’s family provides substantial money or goods to the bride’s family. In return, the bride is expected to uphold tradition and behave accordingly. Therefore, if a *Tongqi* gives birth to a daughter rather than a son and subsequently discovers her husband is MSM, she will receive blame for not producing a male heir [14]. The patrilineal marriage system prevalent in rural China significantly intensifies the stigma endured by *Tongqi*. This system underscores the significance of male progeny for lineage perpetuation, reinforcing societal expectations and entrenched gender norms evaluating a woman’s worth solely by her capacity for producing sons [15]. *Tongqi* are expected to conform to the traditional customs of ancestral reverence, bowing, and kowtowing outside the ancestral hall, a symbolic act to wash off their family’s sins.

*Tongqi* face significant enacted stigma and ostracization within the context of rural China. The cultural emphasis on family honor in China exacerbates this issue, as MSM and *Tongqi* are often perceived as bringing dishonor to their families. The widespread fear of AIDS [16], particularly in rural areas where it is more commonly associated with MSM, further stigmatizes *Tongqi*. Collectively, these factors contribute to the marginalization and stigmatization of *Tongqi* in rural China, often leading to significant mental health implications. Consequently, *Tongqi* grapple with issues such as a lack of sexual intimacy, emotional neglect, feelings of humiliation, and isolation [17]. Efforts to change their bleak circumstances invariably result in societal backlash. *Tongqi* who pursue divorce are construed to have failed as a wife, a mother, and a homemaker. Thus, the patrilineal marriage system considerably amplifies the stigma and challenges faced by *Tongqi*.

### 3.3. Enacted Stigma from Macrostructural Hukou System

For the majority of *Tongqi*, the risk of contracting HIV is higher compared to heterosexual wives in rural China [18,19]. The majority of *Tongqi* are registered with a rural hukou. As stay-at-home wives, all the informants rely on their husband’s income to cover family expenses and typically do not have financial independence. Because their husbands engaged in high-risk sexual activity with MSM, 11 of the 59 *Tongqi* interviewed reported testing HIV positive. All 11 said they contracted HIV from their husbands. In this situation, their rural hukou status poses additional challenges. The hukou determines where one may receive healthcare, but urban hospitals tend to have better care and medications than hospitals serving rural villages. Thus, *Tongqi* rarely have money or access to quality HIV medications, which could alleviate the related symptoms. Yangli (32) lived on the outskirts of Harbin with her 2-year-old and 4-year-old children. She could not find the proper medication for her HIV. She said:

“I need Triumeq (an antiretroviral drug used in China), but to get it, I have to take a 9 h train ride to the city center of Harbin. The rural hospital gave me a cheap substitute, but it only kept my life going a little bit longer. For pain relief, I rely on acupuncture and Chinese herbs. Usually, my body is fine, but sometimes, I have a fever, tiredness, and lethargy. Sometimes, I have swelling or a rash, ulcers or sores in the mouth, and sores on the genitals. The villagers tease me and treat me like a monster.”

Another *Tongqi*, Fangwei (28), contracted AIDS in 2021 and complained of daily discomfort. She said:

“Where do I begin? My health has rapidly declined. My immune system has deteriorated, and even a cold can knock me out. I am always running a fever, sweating at night, and exhausted. I have lost a lot of weight and often have diarrhea. I am prone to infections like pneumonia and even TB. These bugs, no biggie for a healthy person, could be the end of me. The unknown is the worst part—never knowing what is next or how my body will cope. I feel trapped, like I am living in a prison. Even in China’s hot summers, I am freezing. And the fear of dying? It is always there, lurking.”

Another *Tongqi*, Ziwei (31), said she was infected with AIDS from her husband. She said:

“Antiretroviral therapy can help, but it requires strict adherence to a medication regimen, often with unpleasant side effects. If I miss doses, I could develop drug resistance, rendering the medication ineffective. I have been shunned by people who were once friends, even some family members. But you know what is even worse? The way people treat you. Friends who I thought would stick by me turned their backs on me—even some of my family. I have kept my lips sealed, not saying a word to them about it. The loneliness hits you like a ton of bricks. It is like a constant stab in the heart, you know? But I am hanging in there.”

The *Tongqi* are not queer [20,21,22], but many rural villagers think they are. The *Tongqi* are not considered victims caused by marriage fraud by their MSM. The 11 *Tongqi* with HIV reported feelings of desolation, a sense of victimization and self-reproach, depression, anxiety, and fears of societal condemnation as their emotional responses to their diagnosis. In China, an estimated 780,000 individuals live with HIV [23]. The reported rate of HIV cases due to MSM exposure has increased significantly from 0.2% in 2001 [24] to 32.5% in 2009 [24]. In addition, more than 10% out of 13.6 million *Tongqi* infected with HIV through their MSM proactively contact HIV NGOs for HIV/AIDS treatment and medication from the government [25,26].

However, the HIV infection rate among *Tongqi* and its association with the institutional hukou system is often overlooked. Accessing HIV-related medical services in China can lead to discrimination against rural *Tongqi*, as medications and treatments are legally withheld. Since *Tongqi* are perceived to be part of the LGBTQ community, wives with MSM husbands are granted higher status in terms of biological citizenship. The Chinese government maintains control over underprivileged groups within society. Possessing a rural hukou relegates HIV-positive *Tongqi* to the status of marginalized. Some urban residents living with HIV can obtain the antiretroviral drug Triumeq. A monthly prescription of the pills typically costs around CNY 450 (USD 73). However, rural hospitals may not have it in supply, and instead dispense inexpensive substitutes that are ineffective against HIV symptoms. By contrast, urban hukou holders can access superior drug cocktails with multiple pills to combat HIV. Consequently, rural *Tongqi* often receive little or no medical support, while middle-class urban MSM communities receive proper HIV medication and can even travel to the US for treatment. Affluent LGBTQ individuals with HIV have access to various medicines. One method is called Treatment as Prevention (TasP), using antiretroviral therapy (ART) to decrease the likelihood of transmitting HIV. Getting TasP or ART is neither a class issue nor affected by hukou because the medications are difficult to find in China. By reducing the viral load in the patient’s body to undetectable levels, the risk of transmitting the disease to others is significantly reduced [19]. Another method is Pre-Exposure Prophylaxis (PrEP). PrEP refers to the daily intake of HIV medication by individuals who, while not currently infected with HIV, are at risk of contracting the virus, thus, as a preventative measure against HIV infection [19,27,28].

### 3.4. Enacted Stigma by the Government 

Jiaxin, a 30-year-old *Tongqi*, visited her husband’s military camp in 2018 near the Yunnan border after he admitted being an MSM. She wanted revenge because he had repeatedly beaten her. She sought to call him out and ruin his reputation in the camp. However, the camp commander was indifferent and displayed his misogyny with comments that left her disappointed. Jiaxin (30) said:

“The supervisor, a man, denied that my husband was an MSM. He said: “We do not have a gay issue in China; it has never been a social problem here. Leave now, or our security crew will escort you out”. As I left, things got worse. Several security guards threatened to report me to the police for trespassing. I left feeling sad and tearful. I was criticized and bullied by a dozen men. They warned me to stay quiet, stay home, and care for my husband. They said that military men are responsible and heterosexual. Therefore, my husband was a good army man devoted to his family…”

Siyan, a 30-year-old *Tongqi*, has been married for eight years and has a 6-year-old son and a 4-year-old daughter. Her husband works in a government office as a party member. She says he engages in daily acts of deception both at work and in their village. Government offices assume employees are heterosexual. Siyan’s husband repeatedly pressured her to attend an annual gala so they could project the image of a stereotypically wholesome family with a traditional and attractive wife and a charming child. Although Siyan had previously attended the galas, this time, she refused to participate in her husband’s dramaturgy, having been traumatized by his physical and emotional abuse. She said:

“He routinely hit me, pulled my hair, and pushed me into corners, injuring me and filling me with fear. He said this was the only way to ensure I would not endanger his reputation as a good husband, colleague, and staff member in front of his boss and co-workers. He was afraid his colleagues might gossip that he was MSM, and then he would lose face! Therefore, he treated me like a punching bag.”

Zheng [29] contends that some sectors of Chinese society scapegoat male MSM, viewing them as emblematic of “passive masculinity”. Most supervisors from state-owned enterprises do not express concern about the presence of MSM individuals within their ranks, as this aligns with a fundamental policy in the country. China usually ignores *Tongqi* as one of the social problems to maintain societal stability (wenweng 维稳) and encourage the status quo of the marriage between *Tongqi* and MSM.

## 4. Discussions

The socio-cultural values of patrilineal marriage in an extended family in China is important to understand rural *Tongqi* encountered multidimensional stigma in rural China. These socio-cultural values serve as the bedrock of societal expectations and norms, dictating the parameters within which individuals operate. The extended family, in-laws, and fellow villagers, galvanized by these shared cultural values, often become allies in upholding this façade. In rural China, the in-law family members—including both male and female villagers in this study—continue to reinforce heterosexual marriage despite knowing their sons/brothers/residents are MSM. This collective endeavor to maintain societal norms and expectations underscores the powerful influence of socio-cultural values on individual behavior and community dynamics. In rural villages, where gay is stigmatized, gay men are subjected to various forms of stigmatization. As a result, most do not openly disclose their sexual orientation. These men, predominantly local villagers, often succumb to parental pressure to marry and perpetuate their lineage. Their marriages, devoid of a love foundation, are primarily motivated by the desire to establish a family and procreate. The probability of *Tongqi* contracting HIV is higher than that of some heterosexual wives residing in rural villages in China. *Tongqi*, who usually possess rural *hukou*, find it challenging to access medical care from urban China. This impediment exacerbates their struggle to recover from HIV. The ensuing analysis navigates through various layers or dimensions of stigma, providing a systematic reflection on the mutual implications and interactions between these dimensions, culminating in a multidimensional account. Expecting discrimination from medical professionals and family deters queers from public healthcare, heightening susceptibility to death from chronic stress-related pathologies in public institutions due to societal and familial pressures to maintain reputation and interpersonal harmony; this includes military supervisors and cadres in state-owned enterprises [23,30,31]. The lack of supportive legal and social policies further exacerbates workplace discrimination against MSM in a culture that, at times, denies MSM even exists in China [32]. Marrying a *Tongqi* or entering a formal marriage is often seen as a survival strategy within this societal structure (*tizhi* 體制). Goffman’s [33] seminal work underscores that stigma transcends individual experiences, encompassing power dynamics and societal structures. Tyler [34] posits that stigma is a tool wielded by the powerful elite and serves as a mechanism of state coercion. Her research extensively explores the lived experiences of marginalized individuals, examining the intricate ways in which they perceive and present themselves, the tactics they employ to navigate rejection, and the destructive impact of stigma on their interpersonal relationships. Tyler asserts that a comprehensive understanding and subsequent tackling of stigma necessitates an examination of its genesis.

In addition, social structures, including class, gender, wealth, and power, play a pivotal role in shaping societal norms and behaviors to under the enacted stigma of rural *Tongqi*. The most notable manifestations of these societal issues include class-based exploitation and stigma, urban–rural segregation, and gender disparities stemming from the distribution of capital. This comprehensive examination of the *Tongqi’s* experiences within these complex social structures contributes to a deeper understanding of their lived realities, providing valuable insights for future research and policy-making. This study explores the intricate dynamics between *Tongqi* and set against the backdrop of their familial, communal, and bureaucratic interactions, as well as broader national rules and laws, such as the *hukou* system [35]. Additionally, state-based oppression, which arises from the exercise of power in a patriarchal Chinese society, is also a significant concern, despite the inherent challenges and personal sacrifices this may entail. Confucian ethics emphasizes the importance of filial piety to ensure the continuity of the family-kinship system through the production of male offspring. The expanded kinship networks coincided with a greater number of enacted stigma experiences for the *Tongqi*. This cohort of interviewees and their experiences may be representative of other *Tongqi* who are rural, and financially dependent on their husbands. To meet familial and societal expectations of a “normal” life, these MSM individuals are pressured into entering heterosexual marriages, resulting in millions of *Tongqi* living in dire circumstances [36]. Thus, the current hierarchical distribution of power in China favors gendered and heteronormative models of kinship and family, including heterosexual *Tongqi* as “queer” because of their husbands’ sexuality, which denies them the opportunity to fulfill their legitimate heterosexual desires outside marriage [37]. The financial and emotional cost of being an unwilling party in queer kinship cannot be quantified. *Tongqi* often endure emotional distress, social rejection, and discrimination perpetrated by their families and ancestral villages, especially if their husbands have “come out” to their immediate family [26,36,38]. Therefore, the “queer” *Tongqi* are regarded as an underprivileged group living in precarious situations: under pressure from their husbands’ families, villagers, and the government.

Under Chinese law, a man who is sexually involved with another man is not considered guilty of adultery, so a woman involved with such a man has no privilege when seeking custody of children [29]. Most low-educated *Tongqi* wanted to maintain the status quo for fear of losing custody of their children. The most frequently mentioned problem surrounding divorce was the blatantly unequal treatment of the parties. Men are favored in custody battles [39]. Judges routinely deny divorces to women assaulted by their husbands, even when shown medical records of injuries.

The significant transformations in Chinese society since 2020, particularly in the post-COVID-19 era, have been profound to the enacted stigma of rural *Tongqi*. From economic downturns and crises to heightened state intervention in private lives and drastic alterations in family policies, the period under study has borne witness to considerable shifts. Firstly, the fear and uncertainty engendered by the virus have escalated discrimination against marginalized groups, including the *Tongqi* and their gay partners. This has further alienated the *Tongqi*, exacerbating their struggle to seek support and understanding within their communities. Secondly, the economic repercussions of the pandemic have disproportionately impacted rural areas. Numerous rural inhabitants, including the *Tongqi or Tongqi’s* husbands, have either lost their employment or experienced a decline in their income. This economic adversity has imposed an additional layer of stigma, as those facing financial difficulties are often disparaged within their communities. Thirdly, the pandemic has disrupted healthcare services, complicating the *Tongqi’s* access to necessary medical care. This is particularly alarming given the heightened risk of HIV contraction among the *Tongqi* compared to some heterosexual wives in rural villages. Finally, the pandemic has instigated increased state intervention in private lives, leading to intensified scrutiny of the *Tongqi* and their partners. This has further stigmatized them, impeding their ability to live openly and authentically. The challenges in accessing healthcare not only jeopardize their health but also further stigmatize them within their communities.

The extended kinship group and the macrostructure of the government, both of which uphold patriarchy, are identified as hidden contributors to the *Tongqi’s* enacted stigma. The study calls for attention to mediating actors at the community level, such as kinship groups and government offices. It also highlights the paradoxical effects of social networks. While the lack of strong community ties may make the *Tongqi* vulnerable to exploitation and intensify their psychological distress, their social networks can provide much-needed support. The *Tongqi* use their social networks to draw on the collective strength of their sisterhood for mutual assistance and support, providing helpful information and resources. Additionally, promoting the protective efficacy of online social networking benefits from technology upgrades and use by *Tongqi*. They often turn to various individual-based non-governmental organizations (NGOs) for legal advice and counseling. This leads some low-educated *Tongqi* to approach NGOs, such as China Wives of Gay Men Mutual Aid Studio (同妻互援工作室), Little Dust (微尘姑娘同妻救助工作室), and Xiao Delan (小德蘭 Little Teresa) for professional and legal advice. Therapeutic interventions, counseling, and community support can offer a lifeline, helping individuals process trauma, make informed decisions, and rebuild shattered trust.

## 5. Conclusions

Existing scholarship tends to view the mental and social stigma faced by *Tongqi* from an individual perspective, focusing on the *Tongqi* themselves, their husbands, or the *Tongqi*’s HIV and mental illness. This study, however, views enacted stigma as a dynamic social process, constructing masculinity and femininity between the macrostructural forces that contribute to the *Tongqi*’s stigma and the microlevel actions from their families and villagers supporting the traditional patriarchal order. The study rejects a linear connection between gender ideologies and individual action, highlighting instead the various situations and life events that prompt both men and women to mistreat the *Tongqi*. Patrilineal marriage and extended family lineage [40] exacerbate the enacted stigma felt by *Tongqi* and lead to multidimensional stigma, especially after COVID-19.

The 59 low-educated *Tongqi* lacked the necessary skills and resources to break free from the cycle of stigma and abuse. All experienced enacted stigma—particularly the 11 diagnosed with HIV—and encountered numerous hostile and unsympathetic individuals. The 11 *Tongqi* who contracted HIV from their MSM spouses experienced the shock of betrayal followed by anxiety and fear associated with acquiring potentially lethal STIs. However, this did not elicit sympathy from their spouses’ families or local communities. Instead, these *Tongqi* reported a constant vigilance due to the escalating enacted stigma from their spouses’ families, fellow villagers, and larger societal structures. Nevertheless, this stigma’s physical, emotional, and social repercussions require urgent attention and prioritization within international, national, and local public health agendas. This article elucidates the social, political, and personal dimensions of enacted stigma experienced by the *Tongqi*. The empirical evidence underscores the imperative of conceptualizing stigma not merely as an individual phenomenon but as a complex nexus of power dynamics, identity constructs, and societal infrastructures. This research contributes a robust theoretical framework for the operationalization of enacted stigma in scholarly investigations.

The results detailed in this study bear several constraints. Primarily, the *Tongqi* sample was sourced from a specific sample from my personal and professional networks. The MSM samples predominantly comprised primarily male sex workers and individuals engaged in low-skilled jobs in North China. While this convenience sample accurately depicts individuals within this network, the findings cannot be universally extrapolated to the broader *Tongqi* population within China and globally. Future research should strive for a more representative sample, expanding beyond low-skilled jobs to include MSM from various occupations who are married to a *Tongqi*. Second, Chinese-language interviews were translated into English, potentially losing nuance. Third, enacted stigma may persist, but future research should compare stigma with and without HIV and examine the relationship between drug use and sexual activity. Helping those who are vulnerable benefits the general public. Lastly, it is essential to note that this paper solely focuses on enacted stigma, thereby omitting the perspectives of the husbands of the *Tongqi*. This limitation in the scope of the study leaves a significant voice unheard in the discourse.

## Data Availability

Data are contained within the article.
